# Aphids Playing Possum – Defensive or Mutualistic Response?

**DOI:** 10.1007/s10905-018-9662-4

**Published:** 2018-01-18

**Authors:** Aleksandra Bilska, Jacek Francikowski, Aleksandra Wyglenda, Adrian Masłowski, Natalia Kaszyca, Łukasz Depa

**Affiliations:** 1 Department of Zoology, Faculty of Biology and Environmental Protection, University of Silesia, Bankowa 9, 40-007 Katowice, Poland; 2 Students’ Scientific Association of Zoologists “Fauantycy”, Faculty of Biology and Environmental Protection, University of Silesia, Bankowa 9, 40-007 Katowice, Poland; 3 Department of Animal Physiology and Ecotoxicology, Faculty of Biology and Environmental Protection, University of Silesia, Bankowa 9, 40-007 Katowice, Poland

**Keywords:** Thanatosis, immobility, adaptation, fitness, predation

## Abstract

Paper presents the phenomenon of thanatosis or death-feigning in selected aphids species. This specific reaction was predominantly analysed on the example of aphid subfamily Lachninae. Individuals of this group were used in experiments, during which a thanatotic response was induced with various results. The response differed from prolonged thanatosis, lasting for several minutes (*Eulachnus rileyi*), through shrinking behaviour (e. g. in *Stomaphis graffii*) to non-responsive species such as *Cinara* (*Sch*i*zolachnus*) *pineti*. The large interspecies variation of observed responses can be linked to other defensive mechanisms existing in the studied species, as well as to their mutualistic relationship with ants. The behaviour of shrinking is hypothesized to be the mutualistic response, developed from thanatosis, and being adapted to transportation by ant workers.

**Electronic supplementary material:**

The online version of this article (10.1007/s10905-018-9662-4) contains supplementary material, which is available to authorized users.

## Introduction

Thanatosis, or the so called “death feigning” or “playing possum” is a well-recognized phenomenon in many animal taxa (Honma et al. 2006). It is considered as a basic mechanism of the “last chance” defence behaviour, where endangered individual exhibits immobility and catatonic muscle tension. In this state the animal does not lose its conscience and is aware of the phenomena taking place in the environment (Rogers and Simpson 2014). This behaviour, sometimes accompanied by excreting various body fluids, is known both, in vertebrates as well as invertebrates (Miyatake et al. 2004). In case of insects, it is well known in stick-insects or some groups of beetles, where immobility is very common defensive behaviour (Godden 1972, 1974; Prohammer and Wade 1981; Gaiger and Vanin 2006; Miyatake et al. 2008; Farkas 2016).

Apart from various defensive adaptations, aphids are known to exhibit thanatosis, as a form of defence behaviour. It was observed as often exhibited behaviour after fall off the host plant (Losey and Denno 1998; Gish and Inbar 2006; Hodge et al. 2011) by non myrmecophilous aphids. In case of *Acyrthosiphon pisum* the aphids are known to maintain in thanatosis after the drop off for various periods of time, dependent on the nature of the stimulus (Wohlers 1981) and after that they undertake movement of various characters (Niku 1976). The time spent in thanatosis also depends on the height of fall (Niku 1975). This behaviour was also observed in other species such as: *Aualcorthum circumflexum*, *Lipaphis erysimi*, *Macrosiphum euphorbiae* and *Megoura viciae* (Robert 1987), all these species being non myrmecophilous species feeding predominantly on herbaceous plants. However, little is known about the aphid’s thanatosis itself. More important seemed to be the behaviours of waving of abdomens and/or legs, escape and searching of new host plant after drop off as adaptive mechanisms against predators and other environmental factors (Roitberg et al. 1979; Mann et al. 1995; Hodge et al. 2011).

It may be suspected, that in case of apterous morphs of aphids (and contrary to alate morphs), thanatosis could be promoted defensive behaviour in terms of natural selection (Ohno and Miyatake 2007). However, the adaptive nature of this phenomenon is not well understood. Aphids due to their sedentary life mode exhibit a very wide range of defensive mechanisms: waxy excreta, covering the body; alarm pheromones inducing escape behaviour in the colony; mutualistic relationship with ants – which serve as guards protecting aphid colony and even soldier morphs (Dixon 1998). In this view, it seemed interesting to check, whether thanatotic behaviour is somehow correlated with existing defensive mechanisms of aphids.

For this purpose several representatives of the aphid subfamily Lachninae were assigned. This is a relatively homogenous group of aphids in respect of morphology, connected with woody gymnosperms and angiosperms, showing a high variety of ecological adaptations (Jousselin et al. 2013; Chen et al. 2016). We chose this group due to a single field observation of individual of obligate myrmecophilous, tree-trunk feeding species *Stomaphis graffii*, which exhibited remarkable behaviour: after being turned on back and touched with tweezers, it stopped moving and curled appendages towards the body for a few seconds. As we considered at first, it was similar to the so called “pupa-like position” of ants during transportation (Hölldobler and Wilson 1990). We conducted a series of further observations in a laboratory with representatives of related species but with various ecological adaptations. In this paper, we present the extraordinary variability of thanatotic responses in aphids, which, despite a small number of study species, was greater than expected.

## Material and Methods

Despite some aphids being successfully reared, we deliberately focused our study on the subfamily Lachninae as its species exhibit wide range of ecological adaptations, especially mutualistic relations with ants. As a subject of comparison we chose single representatives of Calaphidinae and Aphidinae. All aphids applied in the experiments were acquired from their natural habitat due to serious difficulties with rearing aphids of the subfamilies Lachninae and Calaphidinae as they are tree and woody plant feeders. As the material was collected in autumn, most of the specimens, used in experiments, were oviparous females. All of them were apterous morphs and were adult, which was proven by checking the existence of developed genital plate. Number of individuals studied and number of trials is presented in Table 1. The following list presents the studied species, with additional information relevant to the study:

**Table 1:** Mean values of thanatosis duration in case of species reaction to the stimulus; n – number of individuals studied, N – number of repetitions of measurements

	Mean	SD	Max.	Min.	Mode	Median
*S. graffii* (*n* = 4 *N* = 36)	5.80	1.92	9.73	2.48	6.54	6.38
*M. submacula* (*n* = 8 *N* = 77)	10.94	12.68	65.80	1.48	5.55	6.01
*L. roboris* (n = 8/14 *N* = 78)	5.62	3.98	18.35	1.34	3.22	4.54
*T. troglodytes* (*n* = 5 *N* = 25)	0.82	0.37	1.82	0.55	0.55	0.73
*E. rileyi* (*n* = 7 *N* = 42)	63.90	40.47	179.28	8.41	52.04	57.09
*A. fabae* (n = 5 *N* = 32)	2.69	1.22	5.92	0.89	2.26	2.27
*C. pineti* (*n* = 7 *N* = 39)	–	–	–	–	–	–
*T. annulatus* (n = 7 *N* = 31)	–	–	–	–	–	–

### Lachninae:

*Stomaphis* (*Parastomaphis*) *graffii* Cholodkovsky 1894; collected from *Acer pseudoplatanus*, living with ants *Lasius brunneus*, 09.08.2016–1 apterous viviparous female – original observation – SM1; 23.08.2016–4 apterous viviparous females – applied for experiment.

*Maculolachnus submacula* (Walker 1848); collected from *Rosa* sp., living with ants *Lasius niger*, 18.10.2016–3 apterous viviparous, 5 oviparous females – SM2.

*Lachnus roboris* (Linnaeus 1758); collected from *Quercus robur,* living with ants *Lasius niger*, 25.10.2016–14 oviparous females, all tested, only 8 responsive to stimuli and exhibiting thanatosis – SM3.

*Trama troglodytes* von Heyden 1837; collected from roots of *Artemisia vulgaris*, inside nest of ant *Lasius niger*, 25.10.2016–5 apterous viviparous females (predominant morph in this species, as this species rarely breeds sexually) – SM4.

*Eulachnus rileyi* (Williams 1911); collected from needles of *Pinus nigra,* 18.10.2016–7 oviparous females – SM5.

*Cinara* (*Schizolachnus*) *pineti* (Fabricius 1781); collected from needles of *Pinus sylvestris*, 18.10.2016–7 oviparous females – SM6.

### Aphidinae:

*Aphis fabae* Scopoli 1763; collected from *Gladiolus* x *hybridus,* 18.10.2016–5 apterous viviparous females – SM7.

### Calaphidinae:

*Tuberculatus* (*Tuberculoides*) *annulatus* (Hartig 1841); collected from undersides of leaves of *Quercus robur*, 18.10.2016–7 oviparous females – SM8.

Material for studies was determined basing on key by Blackman and Eastop (2017) and the voucher specimens are deposited in the entomological collection of the Department of Zoology of the University of Silesia in Katowice.

The experiment was carried out about two hours after collecting aphids from their colonies. This time included also the time of aphids transportation to the laboratory. The experiment was conducted during a daylight, between 12:00 and 15:00 PM. The death-feigning behaviour was observed and measured after transfer of single aphid, isolated for ca. 10 min from other individuals (Miyatake 2001), onto a plate surface (diameter of 0.1 m). The death-feigning behaviour was initiated by touching lightly the ventral part of the aphid body with a thin needle or tips of pincers. A single trial consisted of stimulus and provoked immobility which time duration was measured with Behaview software, which allows visualisation of the animal behaviour**.** The time between the stimulus and first sudden and visible movement, usually of the forelegs was counted. Very often, during the induced thanatosis, the hind legs showed constant, but minimal trembling or short, sudden low amplitude movements. It was not taken into account as an exit from the thanatosis state.

The trials were performed under controlled laboratory conditions (temperature ca. 25 °C, 35% of relative humidity**).** A huge effort was made to ensure that aphids were tested in identical environmental conditions and the same procedure was used throughout the experiments.

Aphids behaviour was documented digitally with the Olympus PEN E-PL 6 camera, with lens M.ZUIKO DIGITAL ED 14–42 mm 1:3.5–5.6 II and FOTGA EXTENSION TUBE DG 16 mm FT1 adapter.

## Results

Among the studied species only two: *C.* (*Sch.*) *pineti* and *T. annulatus* were invariably unresponsive to the stimulus and did not exhibit any noticeable state of immobility or thanatosis. The remaining species exhibited various extent and duration of immobility:

### Stomaphis graffii

**Fig. 1:**
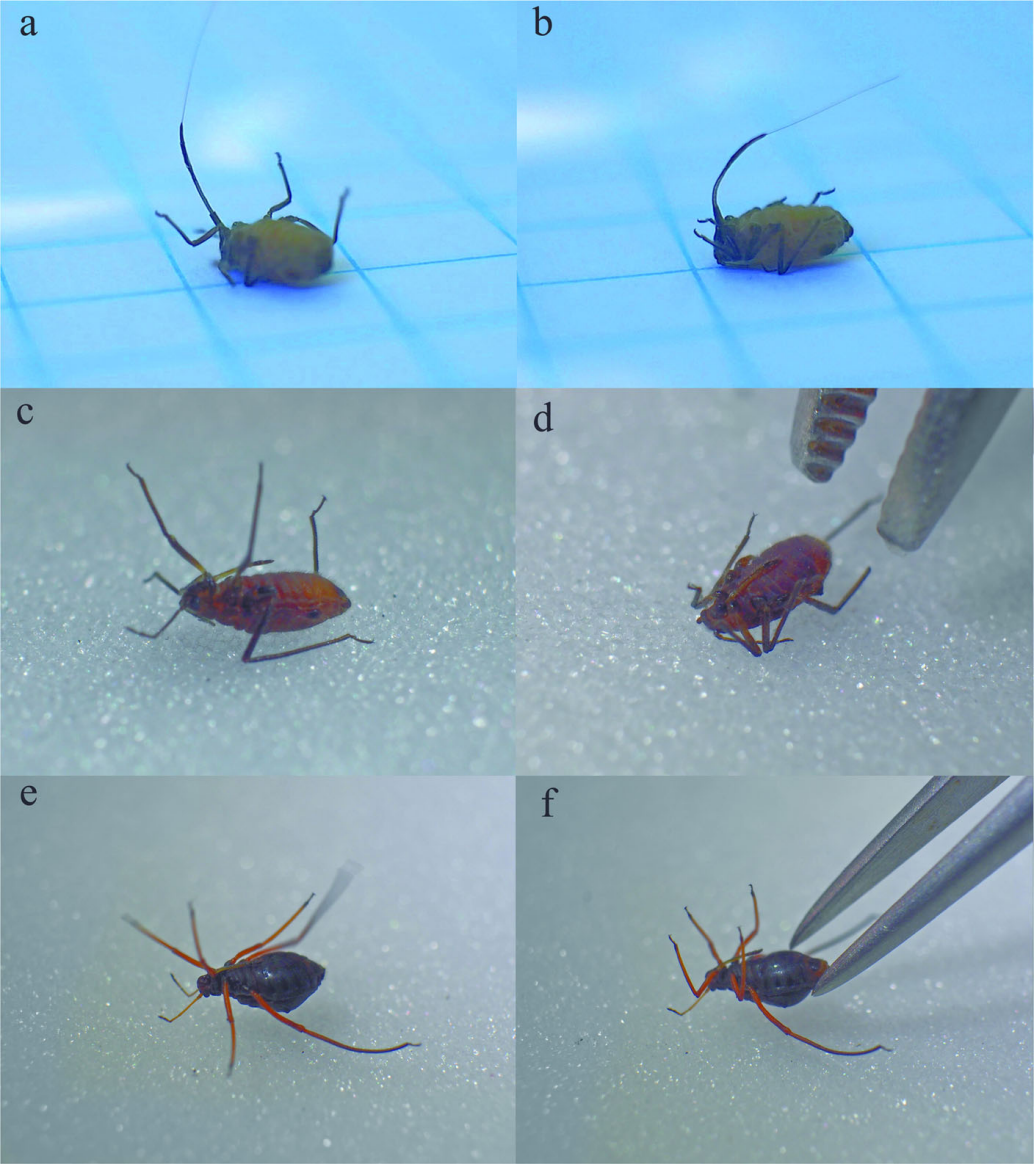
Thanatotic reactions of studied aphids; left – before stimulation, right – after; ab – *S. graffii*, cd – *M. submacula*; ef – *L. roboris*

After turning on back individuals were moving their appendages vigorously trying to turn back (Fig. 1a) (SM1). After touching with a needle, aphids instantly curled their legs towards the abdomen (Fig. 1b). The antennae were put along the body, however, doing slight circular movements with their tips. They stayed in such condition for a few seconds (Table 1.) and then returned to full activity, but were unable to turn to proper position. Presented record (SM1) was done in the field, directly after collection and it gave the reason to provide more detailed studies in the laboratory.

### Maculolachnus submacula

During collection, some of aphids dropped off the plants while some could have been easily collected. During experiment (Fig. 1c), all specimens behaved in the same mode: when touched, they first came to a standstill, only to curl their legs towards their body under continuous touch (Fig. 1d) (SM2). They remained in this condition as long as they were touched, so they could be easily grabbed with pincer and moved or when nudged they remained passive and with legs curled. When the stimulus stopped (Table 1) they returned to full activity in a few seconds and were able to turn to a proper position without difficulties.

### Lachnus roboris

Of 14 collected specimens, which were applied to experiment, only 8 were responsive and could be triggered to exhibit thanatosis. Aphids were very active and they were trying intensely to turn back (Fig. 1e) (SM3). Touching the abdomen with pincer triggered thanatosis with slight curling of legs (Fig. 1f) only for a few seconds and then aphids resumed their activity.

### Eulachnus rileyi

**Fig. 2:**
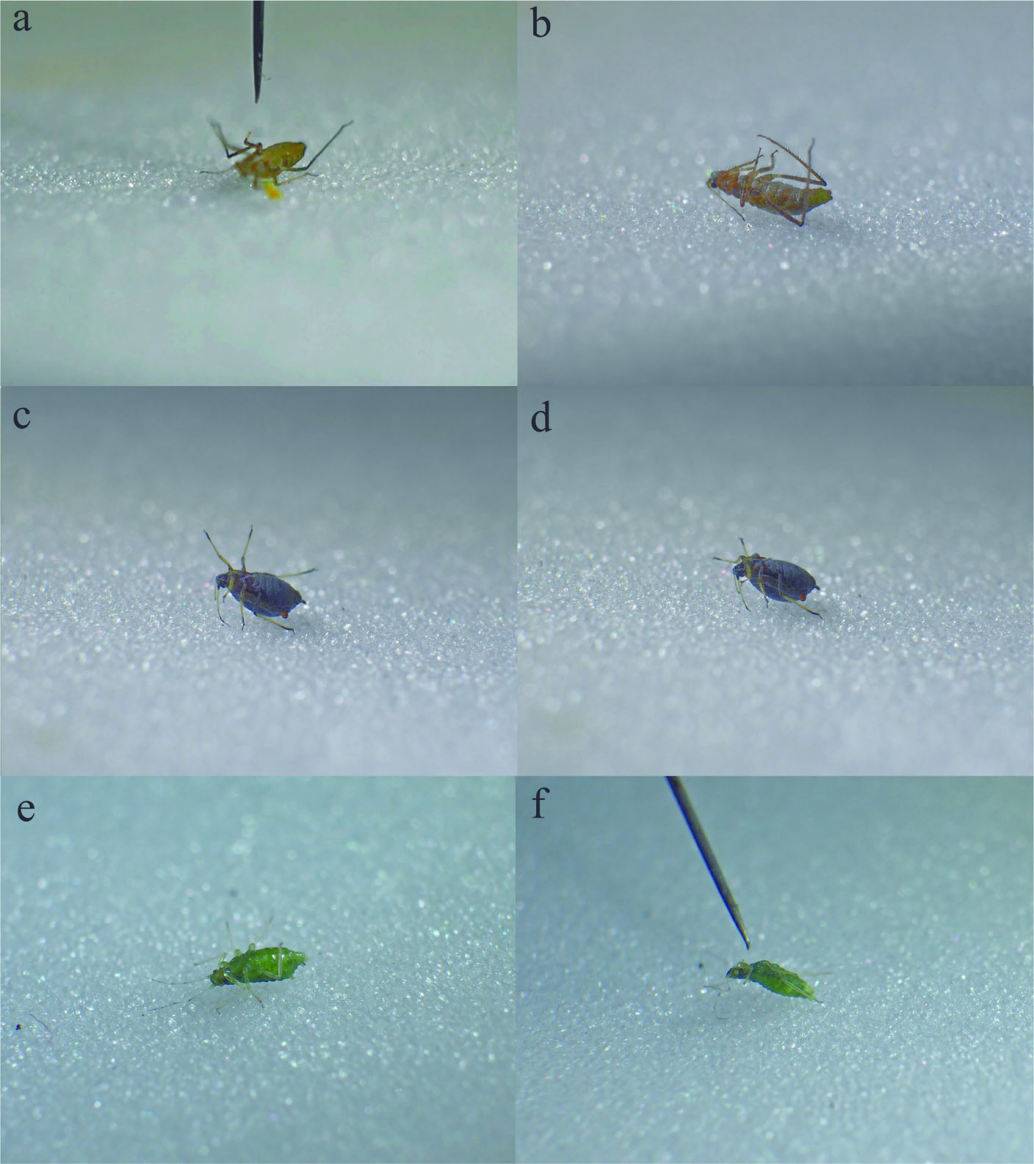
Thanatotic reactions of studied aphids; left – before stimulation, right – after; ab – *E. rilyei*, cd – *A. fabae*; ef – *T. annulatus*

Individuals of this species were very active and were running vigorously so it was difficult to catch them in the field of view of the camera (Fig. 2a) (SM4). However, aphids exhibited thanatosis (Fig. 2b), after being tapped with a needle, which lasted for a significant period of time (Table 1). The appendages were stiff, with only minor trembling of their tips.

### Trama troglodytes

**Fig. 3:**
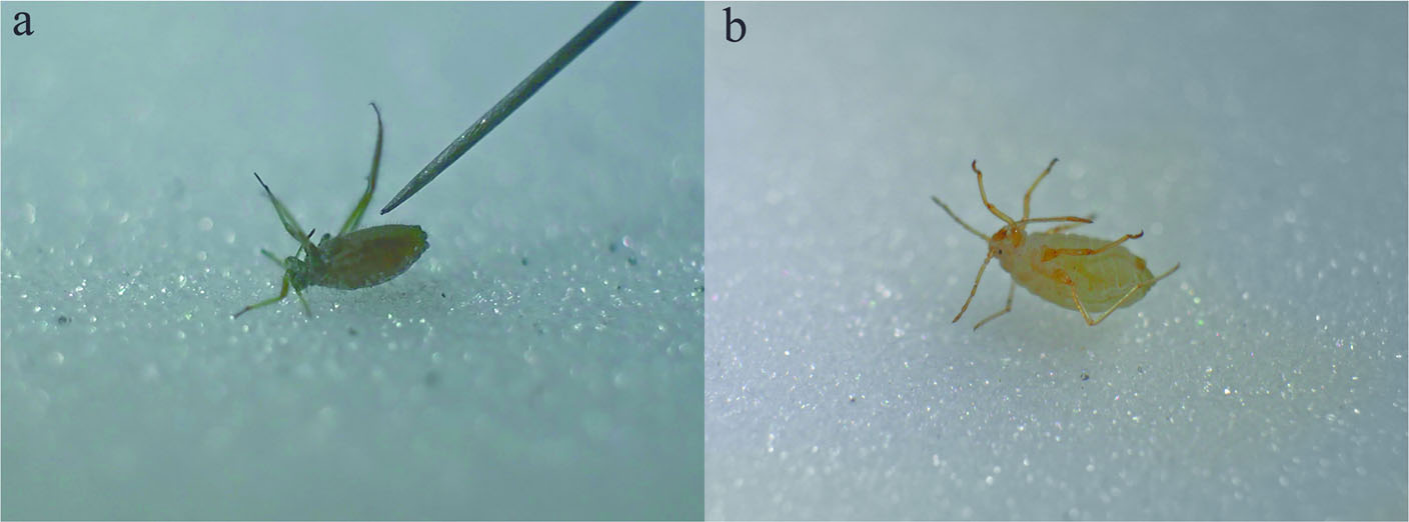
Specimens of aphids non responsive to stimulation: **a** – *C*. (*Sch*.) *pineti*, **b** – *T. troglodytes*

Individuals of this subterranean species did not exhibit thanatosis, except for extremely short immobility after being touched (Fig. 3b) (SM5). Immediately after retracting the needle aphids started to move vigorously and turned back to escape.

### Cinara (Schizolachnus) pineti

Despite touching the aphids with the tips of needle or pincer we could not put individuals of this species into thanatosis (Fig. 3a) (SM6). Aphids were in constant move, trying to turn back and escape.

### Aphis fabae

These aphids could be set into thanatosis by touching with a needle only for short periods of time (Fig. 2c, d) (SM7). During that time their legs were spread and exhibited minor movements.

### Tuberculatus annulatus

Individuals of this aphid species were totally unresponsive to applied stimuli. Neither touching with the needle nor pincers provoked any thanatotic reaction (SM8). On the contrary, aphids were trying to escape with intensity proportional to stimuli and were moving constantly (Fig. 2e). Turned on their backs, they bended whole body vigorously (Fig. 2f) and moved their legs to turn back and run.

As presented in Table 1, the duration of thanatosis varied greatly between the species, even within the single subfamily. The longest period of thanatosis, extending for more than 4 min, was observed in case of *E. rileyi* (SM5), while in case of *T. troglodytes* it mostly lasted for fractions of seconds. The longest time spent in thanatosis in *E. rileyi* during the experiments was almost 3 min, with a mean value exceeding 1 min. *S. graffii* had a relatively stable period of thanatosis, with lowest SD when compared to the mean value (Table 1) while in *M. submacula*, although significant occurrence and well expressed thanatosis, SD was higher than the mean value. Also, in case of *M. submacula*, the mode and median values were half the mean value, due to a few individuals exhibiting thanatosis for longer than half a minute.

## Discussion

From the presented data it seems clear that thanatotic reactions of aphids are more diverse than previously studied (Wohlers 1981) and may be linked to various biological features and ecology of aphids. Such features, which seemed most related to variance in thanatotic response, were presented in Table 2. It seems that the aphids response to external stimuli in our experiments was correlated with the combination of their ecological adaptations rather than purely with the phylogenetic relationships of the aphids tested.

**Table 2:** Biological traits of studied species; thanatosis: 0 - lack, 1- short-term, spread legs, 2 - long-term, 3 – medium-term, pupa-like; h – full cycle, a – permanently parthenogenetic; 0 – without host alternation, 1 – with host alternation; 0 – lack of wax covering, 1 – significant wax covering, 0–1 – only slightly wax powdered; obligatorily (o), facultatively (f) myrmecophilous or (l) not attended; feeding far from ant nest - extranidal, in the close proximity (i.e. in chambers connected with ant nest) – perinidal, inside ant nest – intranidal

	Species	Thanatosis	Biology				Defence mechanisms		Mutualism	
			Life cycle	Host alternation	Host plant	Feeding location	Wax cover	Defence behavior	Degree	Feeding site location
Lachninae	*Stomaphis graffii*	3	h	0	tree	trunk, base	0–1	escape	o	peri-, intranidal
	*Maculolachnus submacula*	3	h	0	tree	trunk, base	0	escape, drop off	o	peri-, intranidal
	*Lachnus roboris*	1	h	0	tree	branches	0	escape, drop off	o	extranidal
	*Trama troglodytes*	0	a	0	herb	roots, base	0–1	escape	o	peri-, intranidal
	*Eulachnus rileyi*	2	h	0	tree	needles	0–1	escape	l	extranidal
	*Cinara (Sch.) pineti*	0	h	0	tree	needles	1	escape	l	extranidal
Aphidinae	*Aphis fabae*	1	h	1	herb	stem/leaves	0	escape, drop off	f	peri-, extranidal
Calaphidinae	*Tuberculatus annulatus*	0	h	0	tree	leaves	0	escape, drop off	l	extranidal

In case of *A. fabae*, we may consider its behaviour as typical “last chance” defensive, thanatotic response, with immobile and spread legs, probably making it difficult for predators to swallow the prey (Honma et al. 2006).

Within single subfamily Lachninae we have the whole spectre of reactions, which seem to depend on the life mode of aphids. A species which seems to have the most primitive, typical kind of response is *E. rileyi* which exhibits thanatosis for a significant period of time. This species feeds on needles of pines, without protection of ants and with a thin wax layer at most and high above the ground level. Additionally, individuals of this species live singly, not in the colonies (Kanturski et al. 2015, 2016). When disturbed, it escapes quickly towards branches or twigs, often falling off the tree. Also the weather conditions may influence their often drop off. In this case, it is not surprise, that aphids casted down by wind very often are adapted to feigning death after the fall, to avoid being treated as a prey. Opposite situation exists in the case of *T. troglodytes*, which lives underground and basically inside the nest of ants (intranidal). When the ant nest is disturbed by a potential predator, ants’ aggressive and protective behaviour seems to be the most significant defence mechanism applied by mutualistic aphids. There is even small need for ants to catch aphids and escape with them, as aphids may instantaneously escape to lower chambers of ant nest, which they actually do. Such opposite life modes may explain opposite reactions of these two species.

*C*. (*Sch*.) *pineti* shows the life mode similar to *E. rileyi*, both species sometimes even live in mixed colonies, but the former is protected by quite significant wax covering which serves as a protection layer against predators and weather conditions. Sometimes colonies encompass the whole needle**.** Probably defence mechanism in the form of dense wax cover and/or better adhesion to a needle is better than dropping off the plant. In this case the thanatotic response was not an adaptive feature undergoing selective pressure and was lost during evolution. Similar case concerns *T. annulatus* which is not covered with wax and lives separately, but on the underside of oak leaves. It seems that dropping off the broadleaved tree provides greater chances of falling on another leaf and hiding under. Also in case of *L. roboris* which lives on twigs and branches of oaks the escape and dropping off can be a good solution – as proven by unresponsiveness of 6 of 14 individuals (Table 1.). Escape is typical behaviour of these species when disturbed. However, in case of *L. roboris* and contrary to *C*. (*Sch*.) *pineti* and *T. annulatus*, it lives in obligatorily mutualistic relationship with ants. Close encounter of dropped off *L. roboris* with ant worker, often of the same species tending it on original feeding location on a tree, brings no harm to aphid. Moreover, it may be moved back to colony of proper feeding location if submissive – thanatotic. In case of *C*. (*Sch*.) *pineti* and *T. annulatus* such encounter may be more risky – quick escape can be better adaptation.

The observed double behaviour of *L. roboris* meets prerequisites for the evolution of an adaptive trait by natural selection expressed by Endler (1986) (Miyatake et al. 2004): variation among individuals and fitness differences. Both traits may be adaptive: thanatosis – either to encounter with ant worker or with predators and active dropping off/escape, in case of danger. On a tree, contrary to intranidal *T. troglodytes*, ants may not be sufficient protection against enemy or other disturbance.

The final case concerns *S. graffii* and *M. submacula* which both present very similar behaviour of curling legs towards the body, resembling pupa-like position of submissive ant workers. Both species are also obligatorily myrmecophilous, often living either in close proximity of ant nest e.g. on a tree or shrub situated directly on the ant nest, in the chambers of soil built around the stems, or inside the nest – at stem or trunk base (Depa 2012; Depa et al. 2012). In these cases aphids face very high possibility of encountering ant worker, yet the escape path to ant nest may not be obvious for a single aphid to walk alone. A further difficulty occurs in *S. graffii*, whose very long mouthparts disables them to be extracted quickly (it usually takes a few minutes) and quick escape. Furthermore, this genus is proven to produce pheromones imitating those of ants tending them (Endo and Itino 2013). In both cases, thanatotic reaction evolved into submissiveness and the trait exhibited partially by *L. roboris* had adaptive significance. It may be also suspected that aphid’s curled legs make the transportation in soil tunnels and chambers easier for ant worker. But we also know, that some aphids, particularly *L. roboris* communicate with ant workers by kick-like movements of their long, hind legs. They do such movements when they are ready to extract honeydew (Hölldobler and Wilson 1990). Perhaps curling legs is also a first communicate for ant workers that it encountered a submissive individual, inhibiting potential aggressive behaviour. After proper recognition by ant, it then allows transportation.

In our opinion, these two kinds of responses resemble two kinds of responses met in anuran amphibians (Toledo et al. 2010) where thanatosis and shrinking were observed. While thanatosis concerned non-toxic species, the shrinking seemed to be either adaptation to being swallowed and spit out after excreting toxins or to avoid further injuries in case of struggling. In aphids, we may consider the behaviour of *S. graffii* and *M. submacula* as shrinking, but being adaptable to mutualism – quick and efficient transportation to a safe place by ant worker.

It seems that the degree of the thanatotic reaction strongly results from strictly ecological adaptations and has undergone the independent development in particular evolutionary lineages in aphid subfamily Lachninae. It seems that two factors influenced its development the most: feeding location and relation with ants. Definitely the initial state was a thanatotic reaction to fall/drop off – similar to *A. fabae* or other non myrmecophilous Aphidinae (Wohlers 1981). It was later either enhanced, when aphids were strongly exposed to fall (*E. rileyi*) or lost, if not (living underground – *T. troglodytes* – although mutualism with ants is definitely involved) or other protective means were applied (*C*. (*Sch*.) *pineti* – wax cover, colonies). When very strict, mutualistic relationship with ants developed, including close proximity of ant nest, the defensive thanatotic response transformed into mutualistic adaptation to transfer by ant worker – shrinking.

Still, however, the subject requires further confirmation with experimental work concerning various morphs as well as various systematic groups of aphids. With a whole set of defensive mechanisms in apterous, sedentary morphs, an extent of variability in thanatotic response may be even larger. It is also important to test, whether in highly polymorphic aphids thanatosis is limited to aptrous morphs only or it occurs also in alate morphs, where fly off could be alternative way of avoiding danger (Ohno and Miyatake 2007).

## Electronic supplementary material

ESM 1 Experiment record of *S. graffii (MP4 3023 kb)*

ESM 2 Experiment record of *M. submacula (MP4 6001 kb)*

ESM 3 Experiment record of *L .roboris (MP4 3971 kb)*

ESM 4 Experiment record of *T. troglodytes (MP4 1480 kb)*

ESM 5 Experiment record of *E. rileyi (MP4 47,029 kb)*

ESM 6 Experiment record of *C.* (*Sch.*) *pineti (MP4 4191 kb)*

ESM 7 Experiment record of *A. fabae (WMV 13629 kb)*

ESM 8 Experiment record of *T. annulatus (MP4 3202 kb)*
